# Muscle Fiber Diameter and Density Alterations after Stroke Examined by Single-Fiber EMG

**DOI:** 10.1155/2021/3045990

**Published:** 2021-08-13

**Authors:** Chengjun Huang, Bo Yao, Xiaoyan Li, Sheng Li, Ping Zhou

**Affiliations:** ^1^Guangdong Work Injury Rehabilitation Center, Guangzhou, China; ^2^Institute of Biomedical Engineering, Chinese Academy of Medical Sciences & Peking Union Medical College, Beijing, China; ^3^Department of Bioengineering, University of Maryland, College Park, MA, USA; ^4^Department of Neurology, Medical College of Wisconsin, Milwaukee, WI, USA; ^5^Department of Physical Medicine and Rehabilitation, University of Texas Health Science Center at Houston, Houston, TX, USA; ^6^University of Health and Rehabilitation Sciences, Qingdao, China

## Abstract

This study presents single-fiber electromyography (EMG) analysis for assessment of paretic muscle changes after stroke. Single-fiber action potentials (SFAPs) were recorded from the first dorsal interosseous (FDI) muscle bilaterally in 12 individuals with hemiparetic stroke. The SFAP parameters, including the negative peak duration and the peak-peak amplitude, were measured and further used to estimate muscle fiber diameter through a model based on the quadratic function. The SFAP parameters, fiber density, and muscle fiber diameter derived from the model were compared between the paretic and contralateral muscles. The results show that SFAPs recorded from the paretic muscle had significantly smaller negative peak duration than that from the contralateral muscle. As a result, the derived muscle fiber diameter of the paretic muscle was significantly smaller than that of the contralateral muscle. The fiber density of the paretic muscle was significantly higher than that of the contralateral muscle. These results provide further evidence of remodeled motor units after stroke and suggest that paretic muscle weakness can be due to both complex central and peripheral neuromuscular alterations.

## 1. Introduction

Stroke is one of the leading causes of death and long-term disability worldwide, which could cause significant structural and metabolic changes in skeletal muscles of the affected limb [1–3]. Electromyography (EMG) studies have been reported to examine paretic muscle changes after stroke, focusing on different levels (muscle fiber, motor unit, muscle, and muscle group) of examination [4]. Among various EMG techniques, single-fiber EMG relies on a special needle electrode with a very tiny recording surface (25 *μ*m in diameter) which allows identification of single-fiber action potentials (SFAPs) [5]. Two parameters are often used for single-fiber EMG processing: fiber density and jitter. Fiber density is defined as the average number of muscle fibers in the recording area (within ~300 *μ*m from the electrode) belonging to one motor unit, which reflects the local organization of muscle fibers within the motor unit. Jitter is the variation in the time interval between the two action potentials of the same motor unit, which provides an indication of the stability of neuromuscular transmission. Both fiber density and jitter are sensitive measures of denervation and reinnervation processes associated with disease or injury.

Single-fiber EMG has been used to examine paretic muscle changes after stroke [6–9]. For example, an increase in fiber density of the abductor digiti minimi muscle was found in the paretic side compared with the contralateral or control group. Fiber density increased at early stage following stroke and thereafter remained stable, indicating that reinnervation already took place in the acute phase of stroke [8]. In another single-fiber EMG study [7], by measuring neuromuscular jitter, it was reported that mean jitters of the extensor digitorum communis and anterior tibial muscles were significantly larger in paretic side than that in normal controls, indicating dysfunction of neuromuscular transmission and an ongoing muscle fiber reinnervation process in the paretic muscle. A recent single-fiber EMG study with the first dorsal interosseous (FDI) muscle of chronic stroke patients indicated little change in jitter although the fiber density was significantly higher in the paretic muscle than that in the contralateral muscle [9].

In addition to fiber density and jitter, the shape of SFAPs also contains valuable information which has not been a focus of analysis in previous single-fiber EMG studies. Larger fibers tend to produce SFAPs with larger peak-peak amplitude and shorter negative peak duration. Based on the SFAP model [10, 11], a simulation study was performed by Zalewska et al., to describe the relationships between SFAP parameters and muscle fiber characteristics [12], from which fiber diameter can be derived from the analysis of SFAP's negative peak duration and peak-peak amplitude. The model was further validated using single-fiber EMG recordings acquired from the frontalis muscle of a healthy subject [13].

In this study, we set to explore central and peripheral neuromuscular alterations after stroke by comparing paretic and contralateral muscles of a group of chronic stroke subjects using single-fiber EMG. It was hypothesized that both central and peripheral neuromuscular changes, including transsynaptic spinal motor neuron degeneration and muscle fiber atrophy, would occur after stroke, which can be captured by single-fiber EMG analysis. More specifically, fiber density was used to assess motor unit loss and compensatory muscle fiber reinnervation. We expected to observe significantly increased fiber density in paretic muscles compared with contralateral muscles, providing evidence of spinal motor neuron degeneration after stroke. Meanwhile, muscle fiber diameter was estimated based on SFAP parameters [12]. We expected to observe significantly reduced fiber diameter in paretic muscles compared with contralateral muscles, providing evidence of muscle atrophy after stroke at the level of individual muscle fibers.

## 2. Methods

### 2.1. Participants

Twelve hemiparetic stroke survivors aged from 45–79 years (5 male, 7 female, age 61 ± 10 years) volunteered to participate in the study. Stroke participants were required to have hemiplegia secondary to an ischemic or hemorrhagic stroke with an onset for more than 6 months and have ability to follow the instructions of the experimenter and move their index finger. In this study, the duration from stroke onset to data collection varied from 1 to 15 years (6.6 ± 4.8 years). Seven subjects had paretic arm in the right side and 5 in the left side. The paretic arm was the dominant arm (before stroke) for 6 of the 12 subjects. The experiment protocol was approved by the Committee for the Protection of Human Subjects at the University of Texas Health Science Center at Houston (UTHealth) and TIRR Memorial Hermann Hospital (Houston, United States). All subjects gave written informed consent before experiment. Maximal pinch and grip forces were measured bilaterally for each stroke subject before EMG data collection.

### 2.2. Experiment Protocols

Subjects sat in a chair with their tested forearm pronated and placed on a height-adjustable table. Prior to electrode placement, the participant's skin above the FDI muscle was scrubbed with alcohol pads to reduce the skin-electrode impedance. A surface reference electrode was placed over the ulna styloid. A Natus UltraPro S100 electromyographic system was used for the study. Single-fiber EMG signals were obtained with a single-fiber EMG electrode (0.45 mm 26 G × 40 mm, Natus Inc.). The single-fiber EMG signals were filtered with a bandwidth of 500–10,000 Hz. The sampling frequency is 48,000 Hz.

To measure fiber density, the electrode was inserted into the FDI muscle and slowly advanced. Subjects were instructed to perform slight FDI voluntary contraction by abducting their index finger to push a 5 kg weight on the table at the horizontal direction. The electrode was optimally positioned to maximize the SFAP amplitude. After optimization, the number of associated single-fiber EMG signals time-locked to the triggering potential (a brief rise time < 300 *μ*s, amplitude > 200 *μ*V) was determined. After each recording, sufficient rest was provided for the subjects to avoid muscle fatigue. The electrode was then advanced to another new position in the FDI muscle, and the process of counting potentials was repeated. For each subject, 20 different recording positions (different depths at different skin insertions) in the midbelly of the FDI muscle were examined. The mean number of potentials counted in all recording sites was calculated as the fiber density.

### 2.3. Fiber Diameter Estimation Model

**Figure 1:**
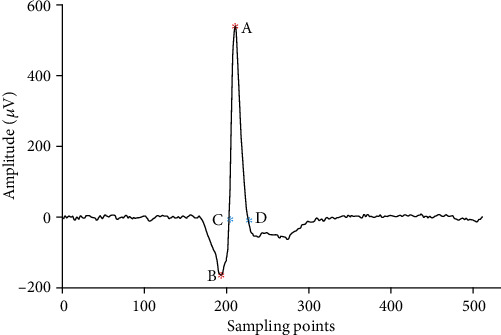
Definition of the SFAP parameters. The peak-peak amplitude is measured from the maximum positive and negative peaks (A to B). The time difference two between zero-crossings (C and D) is the negative peak duration.

The detailed information about the model has been described in previous studies [12, 13]. Briefly, the peak-peak amplitude and the negative peak duration of SFAPs are used to determine the fiber diameter. As shown in Figure 1, the peak-peak amplitude is measured from maximum positive to maximum negative peaks. The time difference between the two zero-crossings, which are following the initial downward and subsequent upward peaks, respectively, is the negative peak duration.

**Figure 2:**
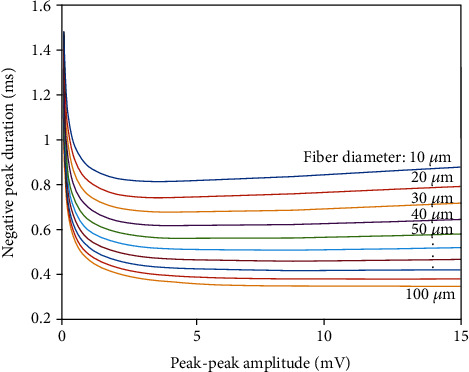
The quadratic relation between the peak-peak amplitude and the negative peak duration under different values of fiber diameters.

The negative peak duration is approximated by a biquadratic polynomial as the following equations:

(1) $$\begin{array}{c} \text{Duration}=F\left(\log_{10}\left(a\right),\text{Diameter}\right), \end{array}$$

where  *a* is the peak-peak amplitude of the SFAP and *F* is expressed as a quadratic function:

(2) $$\begin{array}{c} F\left(x,y\right)=e_{1}+y*\left(e_{2}+y*e_{3}\right), \end{array}$$

where

(3) $$\begin{array}{c} e_{j}=c_{j,1}+x*\left(c_{j,2}+x*c_{j,3}\right),\;j=1,2,3, \end{array}$$

where *y* is the estimated or simulated fiber diameter and *x* is log_10_(*a*). In order to calculate these coefficients, several hundreds of SFAPs were simulated based on the line source model described by Nandedkar and Stalberg [14]. The coefficients *e*_*j*_ are determined by fitting Equations (1) and (2) to the peak-to-peak amplitudes and negative peak durations of the simulated signals. The coefficients given in Table 1 were taken from reference [12]. Based on Equation (1), the dependencies between peak-peak amplitude and negative peak duration under different values of diameter fibers are plotted in Figure 2. The simulated SFAPs of smaller diameters have longer negative peak durations and lower peak-peak amplitudes. The fiber diameters of each subject were estimated using the quadratic model with the data of negative peak durations and amplitudes of the SFAPs recorded in the fiber density session.

### 2.4. Statistical Analysis

Data are presented as mean ± standard deviation (SD). Statistical analysis was performed using SPSS (SPSS, Inc., 2007, Chicago, IL, United States). The Shapiro-Wilk test was used to test for possible deviations from normality. As the fiber density was normally distributed, Student's paired *t*-test was used to investigate side-to-side differences for the fiber density. Cohen's *d* was used as the effect size. Wilcoxon Rank Sum test was used to assess the side-to-side differences for the peak-peak amplitude, negative peaks duration, and fiber diameter, which did not pass the normality test. The effect size was the statistics *z* value divided by square root of observation number. Differences were considered significant when *p* < 0.05.

## 3. Results

For all subjects, the maximum pinch and grip forces (kg) of the paretic side were less than half of the contralateral side. Bilateral recordings of fiber density were successfully completed in all stroke subjects. Fiber density in the paretic muscle was larger than the contralateral muscle in 10 of the 12 subjects. The mean fiber density of the paretic side was significantly higher than the contralateral side (paretic: 1.5 ± 0.2, contralateral: 1.3 ± 0.1, *p* < 0.01, 95%confidence interval = (0.07, 0.37), size effect = 1.22).

**Figure 3:**
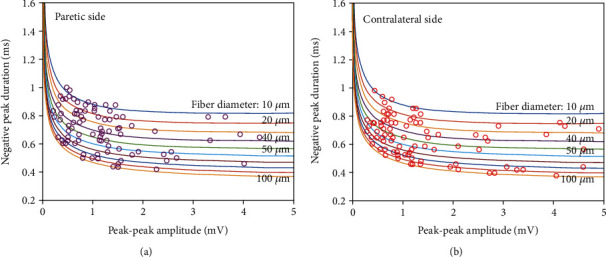
The estimated fiber diameters from paretic and contralateral FDI muscles.

**Figure 4:**
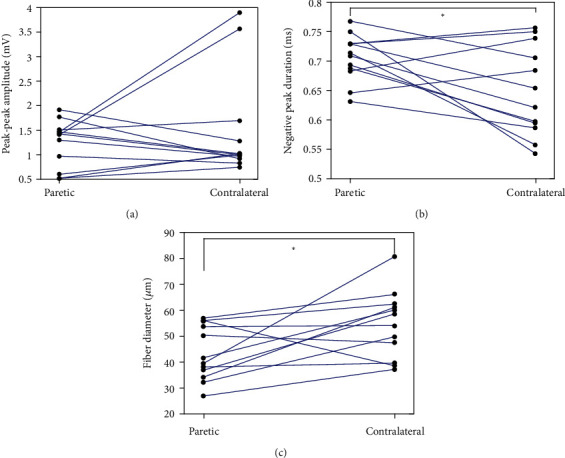
A comparison of (a) SFAP amplitude, (b) SFAP negative peak duration, and (c) estimated fiber diameter between paretic and contralateral muscles of the tested stroke subjects.

In Figure 3, the calculated fiber diameters of the paretic and contralateral FDI muscles are plotted along with the counterlines in Figure 2.  Figure 4 shows the mean SFAP amplitudes, negative peak durations, and estimated fiber diameters of the paretic and contralateral muscles of each stroke subject. The mean SFAP negative peak duration of the paretic muscle was significantly larger than the contralateral muscle (paretic: 0.70 ± 0.14 ms, contralateral: 0.65 ± 0.14 ms, *p* < 0.05, 95%confidence interval = (0.02, 0.10), size effect = 0.2). The mean SFAP peak-peak amplitude was lower in the paretic side compared with the contralateral side, but the difference was not statistically significant (paretic: 1.2 ± 0.8 mV, contralateral: 1.6 ± 1.7 mV, *p* = 0.4). The estimated mean fiber diameter was significantly smaller in the paretic muscle than the contralateral muscle (paretic: 45.3 ± 26.7 *μ*m, contralateral: 53.8 ± 26.8 *μ*m, *p* < 0.05, 95%confidence interval = (−18.49, −2.84), size effect = 0.2).

## 4. Discussion

To assess paretic muscle changes after stroke, different EMG techniques have been applied, e.g., single-fiber EMG, concentric needle EMG, fine wire EMG, macro-EMG, conventional surface EMG, and high-density surface EMG, which can perform different levels of examination from individual muscle fiber to muscle group. The objective of the current study was to assess motor unit alteration and remodeling after stroke by single fiber EMG, focusing not only on fiber density but also on muscle fiber diameter estimated from SFAP parameters.

Muscle fiber diameter provides essential information of a muscle which can be affected by various factors, such as exercise, nutrition, or disease. Increased variability of muscle fiber diameter has been reported in neuromuscular disorders [15]. Reduction in muscle fiber diameters is often associated with myopathies [16]. Muscle biopsy studies indicated decreased muscle fiber diameter for affected muscles after stroke [17, 18]. As the conduction velocity and muscle fiber diameter is positively correlated, changes in muscle fiber diameter can be indirectly assessed by measuring the conduction velocity of propagating action potentials [19–22]. Increased variability in muscle fiber diameter may lead to complex action potential waveforms, broad range of action potential amplitude distribution, and altered global surface EMG parameters [23–27].

In this study, fiber diameters were estimated from the peak-peak amplitude and the negative peak duration of SFAP based on the biquadratic polynomial model [12]. A linear relation between the propagation velocity and the fiber diameter was observed in both healthy individuals and neuromuscular disorder patients [14, 20]. For larger diameter, the time for the potential to pass the electrode is reduced, which results in the shorter negative peak duration. Conversely longer negative peak durations would be associated with smaller fiber diameters. The peak-peak amplitude can also be affected by the fiber diameter. Meanwhile, the distance of the fiber from the electrode plays an important role in determining SFAP amplitude [14]. The peak-peak amplitude will become lower when the electrode is moved further from the muscle fiber. Hence, compared with the peak-peak amplitude, negative peak duration may more accurately characterize the fiber diameter. For the tested stroke subjects, it was found that in the paretic side, the mean negative peak durations were significantly larger than the contralateral side. The peak-peak amplitudes were also smaller for the paretic muscle although not significantly compared with the contralateral muscle. The results indicated that the fiber diameter of the paretic side estimated through the model was significantly smaller than that of the contralateral side. This provides evidence of muscle atrophy at the level of individual muscle fibers. Experimentally, muscle atrophy is often seen in stroke patients due to abnormal neural innervation or long-time muscle inactivity [27].

Fiber density reflects the grouping of muscle fibers of a motor unit within its territory. Reference values of fiber density for a range of muscles were documented in previous literature [28, 29]. For FDI muscle of healthy subjects, one study reported the fiber density should be less than 1.7 [30], which could be significantly increased for patients with motor neuron disease [31–33]. In the current study with stroke subjects, the mean fiber density of the FDI muscle was relatively low compared with previous reports in motor neuron diseases. Consistent with findings from the previous studies of abductor digiti minimi and extensor digitorum communis muscles following stroke [6, 8], fiber density of the FDI muscle was found to be significantly higher in the paretic side than the contralateral side of the tested stroke subjects, suggesting that the paretic muscle had higher focal distribution of the muscle fibers in the motor unit than the contralateral muscle. Previous electrophysiological studies primarily based on motor unit number estimation have reported transsynaptic spinal motor neuron degeneration following stroke, starting as early as initial weeks to months following stroke [24, 34–37]. The degenerated muscle fibers are likely reinnervated by collateral sprouting from intact motor neuron axons, resulting in higher focal distribution of the muscle fibers. Muscle fiber reinnervation may also result in enlarged motor unit territory [38]. Using single-fiber EMG, we examined focal distribution of the muscle fibers in the motor unit. However, information about motor unit territory change was not provided in the current study, which can be further explored using macro-EMG or high density surface EMG techniques [38, 39].

In previous motor unit action potential (MUAP) quantitative analysis of stroke subjects using concentric needle electrodes, it was reported that MUAP parameters (such as amplitude, duration, and complexity) in the paretic muscle were not significantly different between the paretic and contralateral muscles [9, 40]. By recording both single-fiber EMG and concentric needle EMG, it was found that fiber density measures may be more sensitive than MUAP parameters in reflecting changes in the number of muscle fibers innervated by each motor unit, as decreases in muscle fiber diameter will reduce MUAP amplitude, masking increases in amplitude due to increases in the number of innervated muscle fibers. As a result, there might be no significant difference in MUAP parameters between the paretic and contralateral sides although more outliers may be observed from paretic muscles [9, 40].

The FDI is selected as the target muscle, as it is easily accessible for clinical EMG testing and assessment of motor unit properties [41, 42]. Although the maximum voluntary contraction of the FDI muscle was not directly measured in this study, it is likely the paretic FDI muscle had significant weakness given that the maximal pinch and grip strength of the paretic hand was less than half of the contralateral side. Our results indicate that the weakness of the paretic muscle is partly due to muscle atrophy because of reduced fiber diameter or loss of motor units, while muscle fiber reinnervation (as indicated by increased fiber density) can prevent more severe muscle atrophy or weakness that would otherwise occur from motor unit loss. There are other factors that will also contribute to paretic muscle weakness which are not addressed in this study, such as motor unit inactivation or reduced firing rates despite maximum effort as a result of reduced central activation of the muscle poststroke [43–49].

In summary, single-fiber EMG is a useful method to study paretic muscle changes after stroke. A novel feature of the study is a combined examination of both fiber density and fiber diameter to assess the characteristics of voluntarily recruited motor units in chronic stroke subjects. Derivation of muscle fiber diameter from SFAP parameters can provide complementary information to the routine single-fiber EMG analysis. A significant decrease in fiber diameter and a significant increase in fiber density were found in the paretic FDI muscle compared with the contralateral muscle. These findings provide evidence of motor unit remodeling and neuromuscular alterations after stroke.

## Figures and Tables

**Table 1:** The values of the coefficients *c* in Equation (3).

*c*_1,1_	9.5100 × 10^−1^
*c*_1,2_	−2.2321 × 10^−1^
*c*_1,3_	2.0358 × 10^−1^
*c*_2,1_	−7.3845 × 10^−3^
*c*_2,2_	3.4212 × 10^−4^
*c*_2,3_	−1.6564 × 10^−3^
*c*_3,1_	2.5574 × 10^−5^
*c*_3,2_	−3.3074 × 10^−6^
*c*_3,3_	6.4493 × 10^−6^

## Data Availability

All the datasets are available from the corresponding author (PZ) on request.
